# Sorafenib as second‐line treatment option after failure of lenvatinib in patients with unresectable hepatocellular carcinoma

**DOI:** 10.1002/jgh3.12408

**Published:** 2020-08-15

**Authors:** Tetsu Tomonari, Yasushi Sato, Hironori Tanaka, Takahiro Tanaka, Tatsuya Taniguchi, Msasahiro Sogabe, Koichi Okamoto, Hiroshi Miyamoto, Naoki Muguruma, Tetsuji Takayama

**Affiliations:** ^1^ Department of Gastroenterology and Oncology, Institute of Biomedical Sciences Tokushima University Graduate School Tokushima Japan

**Keywords:** hepatocellular carcinoma, lenvatinib, ramucirumab, regorafenib, sorafenib

## Abstract

**Background and Aim:**

Currently, there is no molecular‐targeted agent that has demonstrated evidence of efficacy in patients with unresectable hepatocellular carcinoma (u‐HCC) who have developed resistance to treatment with lenvatinib (LEN). In this real‐world study, we aimed to investigate the therapeutic effect and safety of sorafenib (SOR) in patients with u‐HCC after progression on treatment with LEN.

**Methods (Patients) and Results:**

A total of 13 patients with u‐HCC (12 males and 1 female), who were treated with SOR after progression on LEN, were enrolled in this retrospective study. Therapeutic efficacy was evaluated via contrast‐enhanced computerized tomography at 8 weeks after the initiation of SOR therapy according to modified response evaluation criteria in solid tumors (mRECIST) and RECIST. According to mRECIST, the objective response rate (ORR) and disease control rate (DCR) were 15.3% (2/13) and 69.2% (9/13), respectively. According to RECIST, the ORR and DCR were 0% (0/13) and 69.2% (9/13), respectively. The median progression‐free survival was 4.1 months. The median albumin‐bilirubin scores did not deteriorate significantly at 4, 6, and 8 weeks after initiation of SOR, compared with the scores at the baseline. The most frequent grade 1 or 2 adverse events (AEs) were palmar–plantar erythrodysesthesia, fatigue, diarrhea, and hypertension. There was no incidence of grade 3 AEs.

**Conclusion:**

Treatment with SOR may be effective for u‐HCC after failure on LEN and may not worsen the liver reserve.

## Graphical abstract

We demonstrated that treatment with sorafenib after lenvatinib failure could be useful for unresectable hepatocellular carcinoma. In addition, this treatment strategy may not worsen the liver reserve.

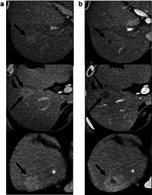

## Introduction

The clinical significance of molecular‐targeted agents (MTAs) in unresectable hepatocellular carcinoma (u‐HCC) has increased drastically since the results from phase III SHARP trial in 2007 were obtained.^1^ A recent phase III REFLECT study demonstrated that lenvatinib (LEN) was noninferior to sorafenib (SOR) as a first‐line systemic therapy for u‐HCC.^2^ Based on these results, several guidelines positioned LEN as a first‐line treatment for u‐HCC in addition to SOR.^3^, ^4^ Furthermore, the opportunities for the administration of LEN in clinical practice have increased.^5^, ^6^, ^7^

In the phase III RESORCE trial, regorafenib (REG) demonstrated survival benefit in u‐HCC as a second‐line systemic therapy in patients progressing on SOR.^8^ The phase III CELETIAL study reported survival benefit of cabozantinib (CAB) in patients with u‐HCC after resistance to SOR.^9^ Recently, the REACH‐2 trial demonstrated that ramucirumab (RAM) showed survival benefit in patients with u‐HCC progressing on SOR and baseline alpha fetoprotein (AFP) level of ≥400 ng/mL.^10^ According to these results, REG, CAB, and RAM were positioned as a second‐line treatment for u‐HCC after the development of resistance to SOR.^8^, ^9^, ^10^

However, there is no evidence of the effectiveness of MTAs in u‐HCC after progression on LEN. Hence, there is an unmet need to search for more effective and less toxic therapeutic approaches as second‐line treatment for u‐HCC after progression on LEN.

LEN is an MTA that targets vascular endothelial growth factor receptors 1–3, fibroblast growth factor receptors 1–4, platelet‐derived growth factor receptor α, RET, and KIT.^11^, ^12^ Conversely, the target genes of SOR are related to angiogenesis such as *VEGFR* and *PDGFR*, as well as *C‐Raf*, wild‐type *B‐Raf*, and mutant *B‐Raf*, which are involved in the MAPK pathway.^13^ Therefore, the target molecules of SOR partly differ from LEN as it mainly targets genes related to angiogenesis. Thus, there is a possibility that SOR can be used as a therapeutic agent after progression on treatment with LEN. In fact, we have reported that LEN treatment is moderately effective as the second‐line treatment after progression on SOR.^14^

Therefore, in this study, we aimed to investigate the therapeutic effect and safety of SOR in patients with u‐HCC after progression on treatment with LEN.

## Methods

### *Patient selection and diagnosis of hepatocellular carcinoma*

This retrospective, observational study evaluated the efficacy and safety of SOR (Nexavar, Bayer Yakuhin Ltd., Osaka, Japan) monotherapy in patients with unresectable advanced HCC after resistance to treatment with LEN. The study was conducted at the Tokushima University between May 2019 and February 2020. This study was approved by the Ethics Committee of Tokushima University Hospital (Approval number; 3489) and was performed in compliance with the 1975 Declaration of Helsinki. The inclusion criteria were based on those of the SHARP trial. Briefly, the antitumor effect was evaluated using conventional response evaluation criteria in solid tumors (RECIST) for measuring treatment responses based on tumor shrinkage, whereas tumor uptake in the arterial phase of contrast‐enhanced computed tomography (CT) was evaluated using modified RECIST (mRECIST)^,^ ^15^, ^16^ an Eastern Cooperative Oncology Group performance status (ECOG PS) score of 0 or 1,^17^ Barcelona Clinic Liver Cancer stages (BCLC) B or C categorizations,^18^ and Child‐Pugh class A. Written informed consent was obtained from all patients. The diagnosis of HCC was based on guidelines established by the Liver Cancer Study Group of Japan.^19^ According to these guidelines, a diagnosis of HCC was confirmed via histology or characteristic radiologic findings, such as typical arterial enhancement of the tumor followed by a washout pattern in the images of the portal venous phase or the equilibrium phase obtained via dynamic spiral CT imaging or contrast‐enhanced magnetic resonance imaging.

### *Treatment with* *SOR*

For patients with no risk factors, we introduced the recommended initial dose of SOR of 400 mg twice daily.^1^, ^20^ The initial SOR dose was reduced by each attending physician according to factors such as bodyweight, age, ECOG PS, and liver function.^21^, ^22^ During SOR treatment, each attending physician decided to reduce the daily dose of SOR according to the grades of adverse events (AEs) or ECOG PS.

### *Measurement of hepatic reserve function*

Hepatic reserve function was assessed according to albumin‐bilirubin (ALBI) grading and Child‐Pugh classification. ALBI grade was calculated based on serum albumin and total bilirubin values, using the following formula: [ALBI score = (log_10_ bilirubin (μmol/L)× 0.66) + (albumin (g/L) × −0.085)], and defined by the following scores: ≤−2.60 = Grade 1, >−2.60 to ≤−1.39 = Grade 2, and >−1.39 = Grade 3.^23^

### *Statistical analysis*

Categorical variables were compared using Fisher's exact test, while continuous variables were compared using Mann–Whitney and Kruskal‐Walls tests. All significance tests were two‐tailed, and statistical significance was set at *P* < 0.05. Kaplan–Meier plots of medians (with 95% confidence interval [CI]) were used to estimate progression‐free survival (PFS) and overall survival (OS). All statistical analyses were performed using Easy R (EZR) version 1.29 (Saitama Medical Center, Jichi Medical University, Saitama, Japan).

## Results

### *Patient characteristics*

A total of 13 patients (12 males and 1 female) with u‐HCC, who were treated with SOR after progression on LEN, were enrolled in this study. Baseline patient characteristics are shown in Table 1. The median age of the patients was 73 years (range, 55–83 years). Three patients were found to be hepatitis B virus antigen‐positive, and four were hepatitis C virus antibody‐positive. The ECOG PS was 0 in nine patients. The median AFP value was 112 ng/mL (range, 1–487 300 ng/mL). There were six and eight patients with Child‐Pugh scores of 5 and 6, respectively. All patients were found to have an ALBI grade 2. SOR was initiated at BCLC stage B and stage C in six and seven patients, respectively. Three patients started SOR with the standard dose (800 mg), while 10 patients started SOR with a reduced dose (400 mg).

**Table 1:** Characteristics of patients with unresectable hepatocellular carcinoma treated with sorafenib

Characteristics	All (*n* = 13)
Age, (years), median [range]	73 [62–81]
Gender (male/female), *n*	11/2
Etiology (HBV/HCV/NBNC), *n*	3/4/6
ECOG PS (0/1), *n*	7/4
Platelets (10^4^/μL), median [range]	11.2 [5.4–30.1]
M2BpGi (C.O.I) [range]	2.51 [0.44–14.6]
Child‐Pugh score (5/6), *n*	6/7
ALBI Grade (1/2/3), *n*	0/13/0
Number of intrahepatic lesions (None/1/2–7/>7)	0/1/8/3
Maximum size of intrahepatic lesion (None/≤50/>50) (mm)	0/10/3
Portal vein invasion (absent/present), *n*	10/3
Extrahepatic spread (absent/present), *n*	11/2
AFP (ng/mL), median [range]	104 [1–487 300]
AFP≧400 (yes/no)	5/8
BCLC stage (B/C), *n*	9/4
Previous treatment times of TAE/TACE [range]	1 [0–4]
Initial dose of sorafenib (800/400), (mg), *n*	3/10

AFP, alpha fetoprotein; ALBI, albumin‐bilirubin; BCLC, Barcelona Clinic Liver Cancer stages; ECOG PS, Eastern Cooperative Oncology Group performance status; HBV, hepatitis B virus; HCV, hepatitis C virus; M2BPGi mac‐2 binding protein glycosylation isomer; NBNC, non‐B non‐C; TACE, transcatheter arterial chemoembolization; TAE, transcatheter arterial embolization.

### *Therapeutic efficacy of* *SOR*

The median observation period after initiation of SOR was 203 (50–335) days. For all the patients, therapeutic efficacy was evaluated using contrast‐enhanced CT at 8 weeks after the initiation with SOR therapy according to mRECIST and RECIST. As per mRECIST, no patient had complete response (CR), two patients had partial response (PR), seven patients had stable disease (SD), and four patients had progressive disease (PD). The objective response rate (ORR) and disease control rate (DCR) were 15.3% (2/13) and 69.2% (9/13), respectively. As per RECIST, no patients had CR and PR, nine patients had SD, and four patients had PD. The ORR and DCR were 0% (0/13) and 69.2% (9/13), respectively (Table 2). The median PFS of all enrolled patients was 4.1 months (95% CI: 2.1–9.2 months; Figure S1, Supporting information). The median OS of 13 patients was not reached. Till date, 76.9% (10/13) of the patients were identified as having radiologic PD after treatment with SOR, of which 80% were treatable after progression on treatment with SOR (REG 60% [6/10], transcatheter arterial chemoembolization 20% [2/10]). Of note, REG was initiated in accordance with the eligibility criteria of the RESORCE study.

**Table 2:** Response to treatment with sorafenib for unresectable hepatocellular carcinoma

Evaluation (mRECIST/RECIST)	mRECIST *n* (%) (*n* = 13)	RECIST *n* (%) (*n* = 13)
Complete response	0 (0)	0 (0)
Partial response	2 (0)	0 (0)
Stable disease	7 (53.8)	9 (69.3)
Progressive disease	4 (30.7)	4 (30.7)
Objective response rate (%)	15.4	0
Disease control rate (%)	69.3	69.3

mRECIST, modified response evaluation criteria in solid tumors.

### *Transition of hepatic reserve function after initiation of treatment with* *SOR*

**Figure 1:**
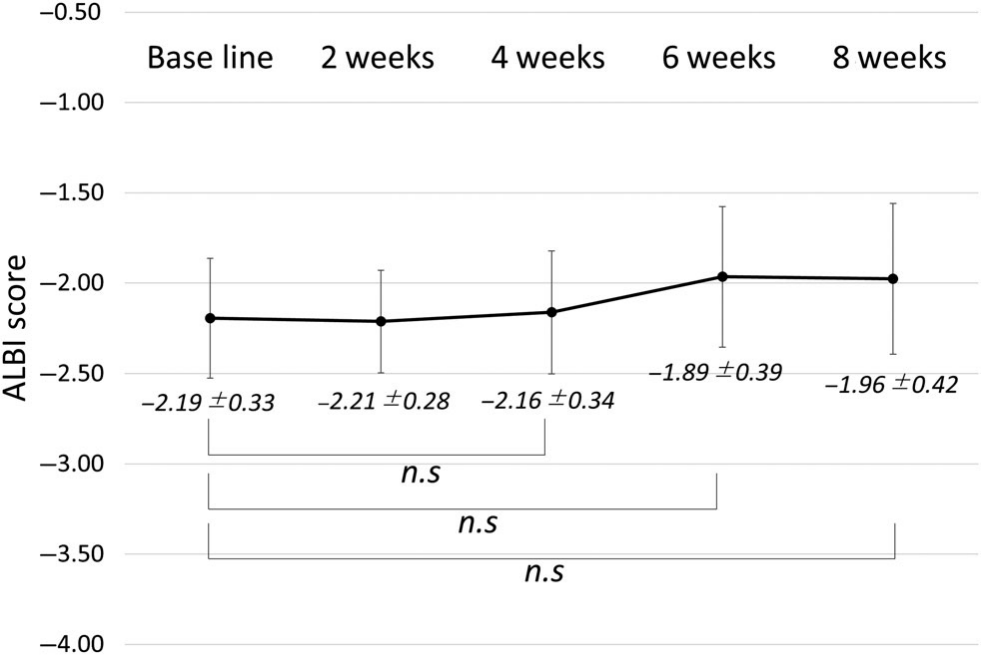
The transition of hepatic reserve function after initiation of treatment with sorafenib at baseline, 2 weeks, 4 weeks, 6 weeks, and 8 weeks. n.s: not significant. ALBI, albumin‐bilirubin

The transition of hepatic reserve function after initiation of SOR for 8 weeks was evaluated in all patients. The median ALBI scores did not deteriorate significantly at 4, 6, and 8 weeks after initiation of SOR (4 weeks: −2.16 ± 0.34, 6 weeks: −1.89 ± 0.39, 8 weeks: −1.98 ± 0.42), compared with the baseline scores (−2.19 ± 0.33) (Fig. 1).

### *Adverse events*

SOR‐related AEs are shown Table 3. The most common AEs were palmar‐plantar erythrodysesthesia (53.8%: 7/13), followed by fatigue (30.7%: 4/13), decreased appetite (30.7%: 4/13), hypertension (30.7%: 4/13), diarrhea (30.7%: 4/13), decreased platelet count (15.4%: 2/13), and dysphonia (15.4%: 2/13). Grade 4 AEs were not observed during the observation period. In addition, there was no discontinuation caused by AEs.

**Table 3:** Adverse events of sorafenib treatment

	All (*n* = 13)				
Event	Any grade	Grade 1	Grade 2	Grade 3	Grade 4
Palmar‐plantar erythrodysesthesia	7 (53.8)	6 (46.1)	1 (7.7)	0 (0)	0 (0)
Fatigue	4 (30.7)	4 (30.7)	0 (0)	0 (0)	0 (0)
Decreased appetite	4 (30.7)	4 (30.7)	0 (0)	0 (0)	0 (0)
Hypertension	4 (30.7)	2 (15.4)	2 (15.4)	0 (0)	0 (0)
Diarrhea	4 (30.7)	2 (15.4)	2 (15.4)	0 (0)	0 (0)
Decreased platelet count	2 (15.4)	2 (15.4)	0 (0)	0 (0)	0 (0)
Dysphonia	2 (15.4)	2 (15.4)	0 (0)	0 (0)	0 (0)
Increased blood bilirubin	1 (7.7)	0 (0)	1 (7.7)	0 (0)	0 (0)
Elevated aspartate aminotransferase	1 (7.7)	1 (7.7)	0 (0)	0 (0)	0 (0)
Elevated alanine aminotransferase	1 (7.7)	1 (7.7)	0 (0)	0 (0)	0 (0)

### *Representative case*

A 77‐year‐old male had been followed up for non‐alcoholic steatohepatitis. The patient was referred to our department for recurrence of multiple HCC. The cirrhosis was classified as Child‐Pugh 5, and the stage of HCC was BCLC stage B.

**Figure 2:**
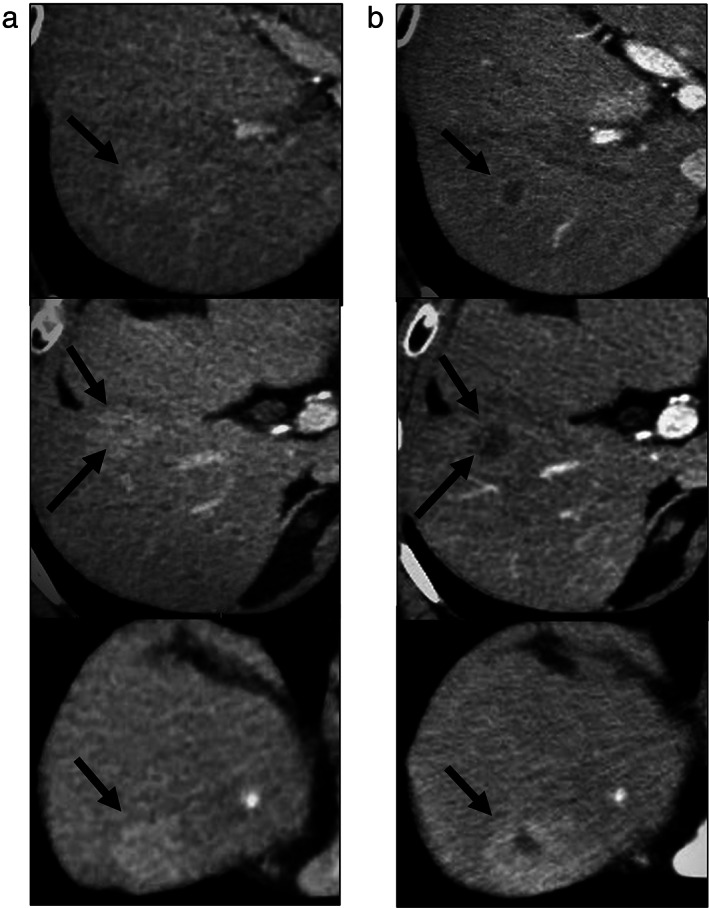
Representative case of a 77‐year‐old male with unresectable hepatocellular carcinoma treated with sorafenib after progression on lenvatinib. (a) A contrast‐enhanced computed tomography examination after lenvatinib failure showed multiple enhanced lesions. (b) Enhancement lesions were decreased at 8 weeks after the administration of sorafenib.

He initially received conventional transcatheter arterial embolization (cTAE). However, contrast‐enhanced CT images, obtained 1 month after cTAE, revealed that lipiodol was washed out from more than half of the HCC nodules. We then started treatment with LEN. After about 12 months of LEN treatment, the patient exhibited PD (according to mRECIST). When the treatment with LEN resulted in PD, the AFP level was ≤400 ng/mL, and hence, RAM could not be used. Therefore, we started treatment with SOR. An enhanced CT examination, performed 8 weeks following SOR initiation, showed decrease in enhanced lesions, and the case was judged as PR by mRECIST (Fig. 2a,b).

## Discussion

In the present study, we showed the therapeutic efficacy and safety of SOR as a second‐line treatment option for u‐HCC after resistance to treatment with LEN. We also demonstrated that, over the duration of treatment with SOR, no significant decrease in ALBI score was observed. To our knowledge, this is the first study to report the feasibility and therapeutic efficacy of SOR after progression on LEN in a clinical setting.

Recently, LEN has been suggested as the first‐line treatment for u‐HCC, in addition to SOR, as per several guidelines. In addition, the opportunities for the administration of LEN in clinical practice have increased.^3^, ^4^ However, there is no treatment option available for patients with u‐HCC after progression on LEN. Therefore, there is a need to search for effective and safe treatment options for patients with u‐HCC who progress on LEN.

In the REACH study, RAM was reported to have significantly extended the survival in cases of AFP >400 ng/mL, as a second‐line treatment, after progression on SOR.^10^ The study reported DCR of 59.9%, and the median PFS of 2.8 months. However, the effectiveness of RAM after progression on LEN has not been fully investigated. Recently, Kuzuya *et al*. reported the results of a small cohort study in which patients with u‐HCC with AFP >400 ng/mL were administered RAM treatment after LEN failure and showed promising therapeutic efficacy and safety, with DCR of 80%, median time to treatment progression of 3.1 months, and the incidence of grade 3 AEs of 10%.^24^

However, considering the fact that, among the patients who progressed after SOR, only 23.3% of patients were eligible for RAM,^25^ it is likely that only a small number of cases could initiate RAM as a second‐line treatment following LEN owing to the strict eligibility criteria (AFP >400 ng/mL). In contrast, when SOR is administered as a second‐line treatment after LEN, there is no need to consider the AFP value, thus suggesting high feasibility of SOR as a second‐line treatment after LEN.

In our study, among the 45 patients who received LEN as first‐line treatment, 27 cases were identified as radiologic PD; 18 patients (66.7%) received subsequent therapies after LEN treatment, including SOR (*n* = 13, 48.1%), RAM (*n* = 3, 11.1%), and transcatheter arterial embolization (TAE) (*n* = 2, 7.4%), while the remaining 9 cases were best supportive care (BSC). Among the 27 cases of radiologic PD, only 29.6% of the patients met the eligibility criteria for the administration of RAM,^10^ thus indicating that a limited number of patients could receive RAM as the second‐line treatment option after LEN.

In the RESORCE study, treatment with REG as the second‐line sequential therapy resulted in a DCR of 65% and median PFS of 3.1 months. The CELETIAL study showed that treatment with CAB resulted in a DCR of 64% and median PFS of 5.2 months. In our study, treatment with SOR resulted in a DCR of 69.3% and median PFS of 4.1 months. Furthermore, although the number of cases was small (*n* = 5), even in AFP >400 ng/mL, which is the eligibility criterion of RAM, DCR was 80%, with a median PFS of 4.1 months. Therefore, our results were comparable to the results of previous studies that reported sequential therapy of MTAs in patients with resistance to SOR. Interestingly, as shown in Figure 2a,b, the enhanced CT examination performed 8 weeks following SOR initiation showed a decrease in enhanced lesions. Several articles have reported that the decreased arterial enhancement during treatment with SOR was related to the OS and could reflect a therapeutic response.^26^, ^27^ Taken together, these results suggest that SOR could be also effective as a second‐line treatment option for u‐HCC.

The drug‐related AEs reported in this study were mostly of grade 1 or 2 severity. Moreover, in line with the results of SHARP and the Asia‐Pacific study, the most frequent AEs in this study were grade 1 or 2 palmar‐plantar erythrodysesthesia, fatigue, diarrhea, and hypertension. In addition, treatment with SOR showed a different AE profile than LEN, and thus, SOR treatment could easily be initiated following treatment with LEN, which suggested that treatment with SOR after progression on LEN was well‐tolerated.

The hepatic reserve function at the initiation of MTA treatment is essential in the treatment of u‐HCC.^28^, ^29^ In the present study, no significant decrease was observed in the ALBI score within 8 weeks after the initiation of SOR. These results suggest that long‐term survival can be expected when using SOR after LEN failure and that it could be easy to subsequence to the third‐line MTAs. Moreover, we could initiate the treatment with REG in 60% (6/10) of the patients after progression on SOR (10/13) as third‐line therapy, which showed the possibility of favorable survival.

The main limitations of our study were its retrospective nature, small sample size, and the short observation period. Therefore, future large‐scale prospective studies are required to confirm the findings of this study.

In conclusion, we demonstrated that treatment with SOR after LEN failure could be useful for u‐HCC. In addition, this treatment strategy may not worsen the liver reserve during treatment.

## Supporting information


**Figure S1.** Progression‐free survival among patients with unresectable hepatocellular carcinoma treated with sorafenib.
