# Supplementary figures and images for: Sorafenib as second‐line treatment option after failure of lenvatinib in patients with unresectable hepatocellular carcinoma
Source: JGH Open. 2020 Aug 15;4(6):1135–9. doi: 10.1002/jgh3.12408 (PMC7731817; doi:10.1002/jgh3.12408)

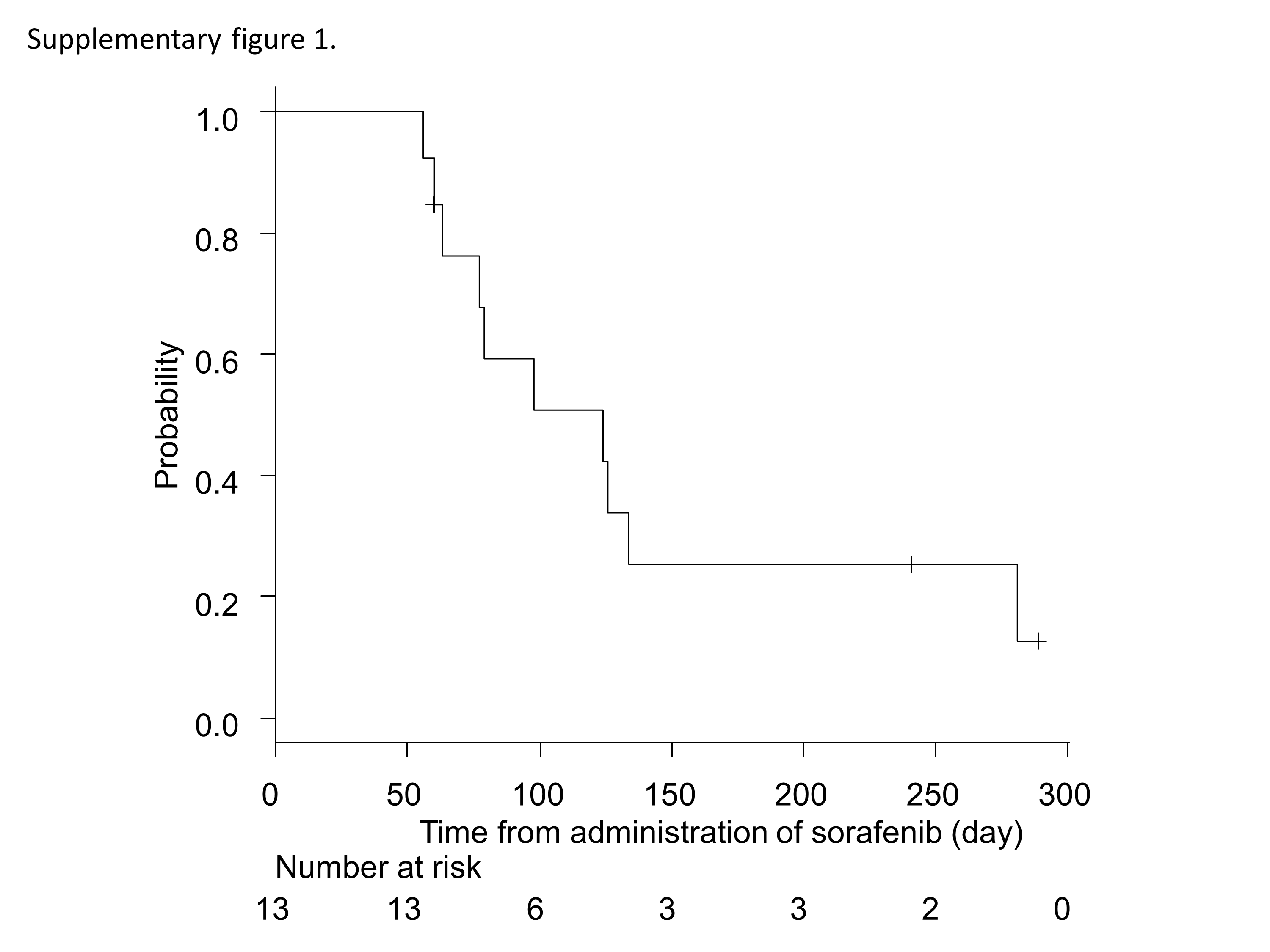

Supplement: Supplementary file 1 — Figure S1. Progression‐free survival among patients with unresectable hepatocellular carcinoma treated with sorafenib. [file JGH3-4-1135-s001.tif]
